# OP21 Advances In The Analysis Of The Impact On Equity In Health Technology Assessment In The Americas Region

**DOI:** 10.1017/S0266462325100792

**Published:** 2025-12-29

**Authors:** Santiago Hasdeu, Jorgelina Alvarez, Laura Lamfre, Anabel Beliera, Andrea Alcaraz, Fernando Argento

## Abstract

**Introduction:**

One of the main objectives of health systems should be to try to reduce inequality gaps. America is the most unequal region in the world. Until recent years, equity continued not to be part of standard health technology assessment (HTA) reports of the main world agencies. We conducted a survey of member institutions of the Health Technology Assessment Network of the Americas (RedETSA) and the Pan American Health Organization (PAHO) to explore the approaches used to measure equity across American countries.

**Methods:**

A survey was conducted addressing equity in HTA in the institutions that make up RedETSA. It was sent to members of the technical teams of the institutions that are part of this network. Through a semi-open questionnaire sent in electronic format, we searched to identify which organizations are addressing equity in HTA, as well as perceptions and personal opinions about its importance and the level of training in this regard. Positive case examples were requested. A scoping review of worldwide methods related to equity-HTA was conducted.

**Results:**

Responses were obtained from 39 individuals working in 34 institutions in 14 countries. All those who responded belong to the technical teams of the institutions that are members of RedETSA. Of these respondents, 97.4 percent consider that the potential impact on equity of new health technologies should always be evaluated in HTA reports, but only 10.8 percent respond that they always do it in their agencies. The approaches used by these institutions were heterogeneous. In order to organize the various proposals that were identified to address equity in HTA, a graph was prepared where the different tools and approaches were classified.

**Conclusions:**

The results of our survey of HTA organizations in the region coincided with global publications, such as the 2015 review of 121 HTA world agencies, which identified that the approach to equity is not systematic; it is performed in few cases and with different methodological approaches. The scoping review identified a wide range of proposed methodological approaches to measure equity.

